# Surface Electromyographic Characteristics of Lower Limb Muscles in Frail Older Adults With Cardiovascular Disease: A Case–Control Study

**DOI:** 10.1002/jcsm.70387

**Published:** 2026-10-05

**Authors:** Pingping Huang, Shiqi Chen, Linjing Wu, Jiahua Li, Rongxiang Zhang, Shufen Liu, Yuan Chen

**Affiliations:** ^1^ Department of Nursing Xiamen Cardiovascular Hospital of Xiamen University, School of Medicine, Fujian Branch of National Clinical Research Center for Cardiovascular Diseases Xiamen China; ^2^ School of Nursing Fujian University of Traditional Chinese Medicine Fuzhou China; ^3^ Department of Cardiology Xiamen Cardiovascular Hospital of Xiamen University, School of Medicine, Fujian Branch of National Clinical Research Center for Cardiovascular Diseases Xiamen China

**Keywords:** cardiovascular disease, frailty, lower limb, older adults, surface electromyographic (sEMG)

## Abstract

**Background:**

Frailty predicts poor outcomes in older adults with cardiovascular disease (CVD) and is closely related to neuromuscular dysfunction. Lower limb surface electromyography (sEMG) during walking may capture frailty‐related motor alterations, but its characteristics and discriminative value in older adults with CVD remain unclear. This study aimed to compare lower limb sEMG features between frail and nonfrail older adults with CVD and evaluate their ability to identify frailty.

**Methods:**

This case–control study included 150 older adults with CVD (75 frail, 75 nonfrail; 80 men, 70 women). Frailty was defined using the Fried frailty phenotype. Wireless sEMG signals were recorded bilaterally from the rectus femoris, semitendinosus, tibialis anterior and medial gastrocnemius during a standardized 10‐m walk test. Time‐domain parameters, frequency‐domain parameters, symmetry indices, contribution ratios and activation‐sequence rank were compared with false‐discovery‐rate correction. Binary logistic regression and receiver operating characteristic (ROC) analysis were used to identify factors independently associated with frailty and evaluate discriminative performance.

**Results:**

Frail participants were older (76.04 ± 7.57 vs. 68.55 ± 6.09 years), more frequently exposed to polypharmacy (93.33% vs 70.67%), required longer to complete the 10‐m walk test (13.34 ± 3.23 vs. 9.44 ± 1.21 s) and had lower Tinetti Performance‐Oriented Mobility Assessment (POMA) scores (21.51 ± 4.27 vs. 27.59 ± 0.92; all *p* < 0.001). The frail group showed higher median integrated EMG (IEMG) of the left semitendinosus (534.4 [451.3, 680.7] vs. 455.5 [384.5, 542.0] μV·s, FDR‐adjusted *p* = 0.0084) and higher activation‐sequence rank of the left rectus femoris (4.333 [3.000, 5.667] vs. 3.333 [2.333, 4.333], FDR‐adjusted *p* = 0.0404). In the frequency domain, median frequency (MF) was higher in the right rectus femoris, bilateral tibialis anterior and right medial gastrocnemius, whereas mean power frequency (MPF) was higher in six muscles (all FDR‐adjusted *p* < 0.05). In multivariable analysis, MF of the right rectus femoris (OR = 1.067, 95% CI 1.003–1.135, *p* = 0.038), MF of the right medial gastrocnemius (OR = 1.054, 95% CI 1.009–1.102, *p* = 0.019), activation‐sequence rank of the right tibialis anterior (OR = 0.640, 95% CI 0.448–0.914, *p* = 0.014), age (OR = 1.128, 95% CI 1.044–1.218, *p* = 0.002) and Tinetti POMA score (OR = 0.784, 95% CI 0.669–0.918, *p* = 0.003) were independently associated with frailty. The sEMG + age model achieved an area under the curve (AUC) of 0.8418 (sensitivity = 0.8933; specificity = 0.6400), approaching the full model including Tinetti POMA score (AUC = 0.8740; sensitivity = 0.9200; specificity = 0.7067).

**Conclusions:**

Lower limb sEMG features during walking were independently associated with frailty in older adults with CVD. The simplified sEMG + age model showed good discriminative performance and may provide candidate objective markers to complement conventional frailty assessment.

## 1 Introduction

Cardiovascular disease (CVD) remains the leading cause of death among older adults globally [1]. Frailty, a common geriatric syndrome marked by multisystem physiological decline—including neuromuscular, metabolic and immune dysfunction—substantially increases vulnerability to stressors. It is characterized by reduced muscle strength, endurance and functional reserve and is closely linked to poor outcomes such as falls, disability and mortality [2, 3]. Frailty and CVD are strongly interrelated. The prevalence of frailty in older adults with CVD is markedly elevated, affecting up to 30% of those with coronary artery disease, 80% with heart failure and 74% with aortic stenosis [4]. Moreover, frail individuals with CVD experience higher risks of mortality, bleeding and adverse cardiovascular events, even at early disease stages [4, 5]. The relationship is bidirectional: CVD may accelerate frailty progression, whereas frailty can exacerbate cardiovascular decline, forming a self‐perpetuating cycle [6, 7]. Frailty is dynamic and potentially reversible; its progression increases cardiovascular risk, whereas timely intervention may reduce it [8]. Early identification and individualized management of frailty in older adults with CVD may therefore be essential for improving clinical outcomes.

Sarcopenia is widely recognized as a fundamental biological substrate of frailty [9]. Age‐related declines in muscle strength, mass and function—particularly in the lower limbs—contribute to impaired mobility and increased frailty risk in older adults [10, 11]. Under normal conditions, muscle homeostasis is maintained through a tightly regulated balance of protein synthesis and degradation, modulated by neural, endocrine and immune systems, as well as nutritional and physical activity status. In frailty, this balance is disrupted, accelerating sarcopenia and functional decline [12]. Importantly, muscle weakness and wasting may reflect structural and functional deterioration at the neuromuscular junction (NMJ) [13]. Clark [14] suggests that strength loss with age is more closely linked to impaired neuromuscular activation than to atrophy alone. Emerging evidence further indicates that frailty is associated with specific electrophysiological alterations in skeletal muscle, which may precede clinically detectable weakness [15, 16]. Surface electromyography (sEMG), a noninvasive and sensitive tool for capturing muscle activation patterns, can detect subtle neuromuscular dysfunction and offers dynamic insight into motor control strategies [17, 18]. sEMG may thus serve as a promising adjunct for the early detection of frailty, particularly in older adults with cardiovascular disease.

Gait performance is a key indicator of frailty, with impaired walking ability often reflecting underlying neuromuscular dysfunction and reduced mobility [19, 20, 21]. In older adults with cardiovascular disease (CVD) and coexisting frailty, muscle recruitment patterns during gait may mirror real‐world motor performance. Among frailty‐related features, gait speed shows the strongest association with both all‐cause and cardiovascular mortality, making it a robust prognostic marker [22]. Maximum gait speed, in particular, independently predicts outcomes in CVD patients and, when combined with usual gait speed, enhances risk stratification accuracy [23]. Gait speed has demonstrated predictive value comparable to the 6‐min walk test but offers greater simplicity and feasibility in clinical practice [24]. The 10‐m walk test (10MWT), widely used for its brevity and reproducibility, is especially suitable for patients with limited capacity or space constraints. During walking, coordinated activation of lower limb muscles—including the rectus femoris, semitendinosus, tibialis anterior and gastrocnemius medialis—is essential for maintaining posture and propulsion [25, 26]. sEMG recordings during the 10MWT enable dynamic assessment of these activation patterns, providing insight into neuromuscular function in frailty.

Accordingly, this study was designed to collect and compare surface electromyography (sEMG) characteristics of major lower limb muscles during a standardized 10‐m walk task in frail and nonfrail older adults with cardiovascular disease. The objective was to elucidate differences in neuromuscular control associated with frailty and to generate empirical evidence that supports its early identification, objective evaluation and mechanistic investigation in this high‐risk population.

## 2 Methods

### 2.1 Study Design

This observational case–control study was conducted in accordance with a previously published protocol in PLOS ONE [27] and reported following the Strengthening the Reporting of Observational Studies in Epidemiology (STROBE) guidelines. The study adhered to the principles of the Declaration of Helsinki and was approved by the Ethics Committee of Xiamen Cardiovascular Hospital, Xiamen University (Approval No. [2023] Medical Ethics Committee No. 39). All study procedures were implemented as planned, with a minor clarification of the inclusion criteria to reflect the actual recruitment setting.

### 2.2 Study Population and Grouping

This study was conducted between November 2024 and June 2025 in both the outpatient and inpatient departments of Xiamen Cardiovascular Hospital, affiliated with Xiamen University, in accordance with a previously published protocol [27], which specified this hospital as the sole recruitment site and included adults aged ≥ 60 years meeting either frailty or nonfrailty criteria. As all participants were recruited from this cardiovascular specialist hospital and were clinically diagnosed with cardiovascular disease (CVD), the formal study refined the inclusion criteria to explicitly require confirmed CVD, thereby enhancing sample homogeneity and clinical relevance. This refinement represents a clarification aligned with the original protocol and recruitment setting, rather than a protocol deviation.

Frailty status was assessed using the Fried frailty phenotype. Participants meeting the frailty criteria were assigned to the frail group, whereas those not meeting the criteria were classified as nonfrail. Sample size was estimated based on a pilot effect size (Cohen's *D* = 0.433) using G*Power 3.1. To achieve 80% power with a two‐tailed α of 0.05, at least 67 participants per group were required. Allowing for a 10% attrition rate, the final target sample size was 75 per group, totalling 150 participants. All participants provided written informed consent after receiving a detailed explanation of the study.

### 2.3 Inclusion and Exclusion Criteria

Participants were eligible for inclusion if they met the following criteria: (1) aged ≥ 60 years; (2) confirmed diagnosis of cardiovascular disease (CVD), as defined in the 9th edition of Internal Medicine and supported by imaging (e.g., CT or MRI), including coronary artery disease, heart failure, hypertension, arrhythmias, cardiomyopathies or valvular heart disease; (3) ability to ambulate independently without assistive devices; (4) intact reading comprehension, cognitive function and verbal communication sufficient to complete study assessments; (5) voluntary participation with written informed consent; and (6) classification as either frail (meeting ≥ 3 criteria of the Fried frailty phenotype) or nonfrail (meeting none of the criteria) [28].

Exclusion criteria were as follows: (1) severe neuromuscular or musculoskeletal disorders; (2) unstable or severe cardiovascular, cerebrovascular or psychiatric conditions that could compromise safety or assessment completion (e.g., decompensated heart failure, acute myocardial infarction or stroke within the past 3 months or acute psychiatric episodes); (3) inability to cooperate with study procedures or perform physical tasks; (4) known allergy to surface electrodes; and (5) other serious medical conditions or investigator‐determined factors precluding safe and complete participation.

### 2.4 Experimental Procedures

All eligible participants underwent testing in a dedicated electromyography laboratory maintained at a constant temperature of 25°C, following provision of written informed consent.

#### 2.4.1 Equipment Preparation and Electrode Placement

Surface EMG signals were recorded using a wireless acquisition and feedback system (SG‐800A, Zhejiang Dno Medical Technology Co. Ltd., China) with a sampling rate of 2000 Hz. Bilateral recordings were obtained from the rectus femoris, semitendinosus, tibialis anterior and medial gastrocnemius. Before electrode placement, hair at each site was shaved and the skin cleaned with 75% alcohol. Disposable surface electrodes (E5, Zhejiang Dno Medical Technology Co. Ltd.) were positioned on the centre of the muscle belly, aligned parallel to the muscle fibres, following Surface Electromyography for the Non‐Invasive Assessment of Muscles (SENIAM) guidelines. A reference electrode was affixed to a noncontractile area. All electrodes were secured with elastic bandages to minimize motion artefacts. A representative photograph of electrode placement and fixation is presented in Figure S1.

#### 2.4.2 10‐m Walk Test

Participants completed a standardized 10‐m walk test along a 15‐m straight indoor walkway, including 1‐m acceleration and deceleration zones at each end. Following electrode placement and signal stabilization (< 10 μV), participants were instructed to walk at their usual pace. Dynamic sEMG signals were synchronously recorded, and walking time was measured using a stopwatch. Each participant performed three trials with 1‐ to 2‐min rest intervals; the average time was used for analysis. Walking aids were not permitted. A trained investigator accompanied each participant during testing to ensure safety without providing physical assistance.

### 2.5 Data Processing

Surface electromyography (sEMG) signals were processed using proprietary software integrated with the wireless sEMG analysis system (Zhejiang Dno Medical Technology Co. Ltd., China). To reduce gait‐related artefacts at initiation and termination, data from the fourth to eighth gait cycles were selected for analysis. Preprocessing involved a two‐stage filtering protocol. First, a zero‐lag Butterworth band‐pass filter (20–500 Hz) was applied to attenuate environmental and nonmyogenic noise. Second, ECG artefacts were suppressed using an adaptive algorithm based on signal statistical characteristics. Representative raw sEMG waveforms captured during walking are shown in Figure S2.

Filtered signals were visually inspected by trained analysts to exclude segments with motion artefacts or excessive noise. After artefact inspection, valid segments were full‐wave rectified and smoothed using a moving‐average filter. Time‐domain parameters were then calculated from the processed signals using the proprietary software integrated with the wireless sEMG analysis system and were analyzed as task‐derived sEMG measures obtained during the standardized 10‐m walk test. For spectral analysis, smoothing was omitted to preserve spectral fidelity, and the corresponding frequency‐domain parameters were calculated from the power spectral density of the filtered, unsmoothed signals. Maximal voluntary contraction (MVC) normalization was not performed because the study population consisted of older adults with CVD, including frail individuals, for whom maximal contraction testing may be difficult to standardize and may raise safety and tolerability concerns. Accordingly, time‐domain parameters were interpreted as walking task‐derived sEMG measures rather than as %MVC‐normalized activation levels.

### 2.6 Outcome Measures and Calculation Methods

#### 2.6.1 Primary Outcome Measures

The primary outcomes included three time‐domain electromyographic parameters, two frequency‐domain parameters and three derived indices: symmetry index (SI), muscle activation‐sequence rank and muscle contribution ratio. All metrics were calculated using proprietary software based on sEMG signals from the fourth to eighth gait cycles during the 10‐m walk test. Definitions, units and calculation methods are described below.

1 Time‐domain parameters

Time‐domain features comprised root mean square (RMS), integrated EMG (IEMG) and average EMG (AEMG). RMS and AEMG were expressed in microvolts (μV), whereas IEMG was expressed in microvolt‐seconds (μV·s). These parameters were calculated from the rectified and smoothed EMG signals using the following formulas:

$$\begin{array}{c} \mathrm{RMS}=\sqrt{\frac{\sum_{i=0}^{N}\text{Data}{\left[i\right]}^{2}}{N}},\;\mathrm{IEMG}=\sum_{i=0}^{N}\left|\text{Data},\left[i\right]\right|\times \Delta t,\\ \mathrm{AEMG}=\frac{1}{N}\sum_{i=1}^{N}\left|\mathrm{xi}\right|. \end{array}$$

Specifically, *Data*[*i*] denotes the EMG value at the *i*th sampling point, with *N* representing the total number of data points acquired within the analysis window. The index *i* ranges from 1 to *N*, and *Δt* refers to the time interval between adjacent sampling points. *xi* denotes the absolute EMG value at point *i*.

2 Frequency‐domain features

Frequency‐domain parameters included mean power frequency (MPF) and median frequency (MF), both expressed in hertz (Hz). These parameters were computed from the power spectral density (PSD) of the filtered, unsmoothed EMG signals using the following formulas:

$$\mathrm{MPF}=\frac{\sum_{i=1}^{N}f_{i}\cdot P_{i}}{\sum_{i=1}^{N}P_{i}},\mathrm{MF}=\text{Median}\left(f\left(P\left(f\right)\right)\right)$$

Here, *f*_*i*_ refers to the *i*th frequency point, *P*_*i*_ to the power spectral density at *f*_*i*_ and *N* to the total number of frequency points. *P*(*f*) refers to the PSD expressed as a function of continuous frequency *f*, and *f* represents a specific frequency value within the signal spectrum.

3 Symmetry Index (SI)

The SI quantifies the bilateral symmetry of muscle activation and was calculated as SI = (RMS_left − RMS_right) / (RMS_left + RMS_right). SI was treated as a dimensionless index. A positive SI indicates greater muscle activation (in terms of RMS amplitude) on the left side, whereas a negative SI reflects dominance on the right.

4 Muscle activation‐sequence rank

Muscle onset was defined as the first instance at which the average EMG amplitude across 10 consecutive samples exceeded 1.7 times the peak value of the initial 20 baseline samples. Offset was marked when the average subsequently fell below this threshold. For each gait cycle, the eight recorded muscles were ranked according to their onset times, and each muscle's activation‐sequence rank was averaged across the analyzed gait cycles. This variable was treated as a unitless ordinal rank, with larger values indicating later activation order within the gait cycle.

5 Muscle contribution ratio

Muscle contribution ratio was defined as the percentage of each muscle's IEMG relative to the total IEMG across all eight recorded muscles over the analyzed gait cycles. This metric reflects the proportional contribution of individual muscles to the overall neuromuscular output during walking [29].

#### 2.6.2 Secondary Outcome Measures

Secondary outcomes were collected to characterize mobility and balance performance associated with Fried phenotype‐defined frailty and included average walking time and functional assessments of gait and balance. Each participant completed three trials of the 10‐m walk test; the mean duration was calculated to reflect basic ambulatory performance. Following sufficient rest, a trained assessor—certified through standardized instruction—administered the modified Tinetti Performance‐Oriented Mobility Assessment (POMA) to evaluate gait and balance. The modified POMA, adapted by Cobbs et al. from the original version, comprises two subscales: balance (9 items, maximum score 16) and gait (7 items, maximum score 12), with a total score ranging from 0 to 28. Higher scores denote better gait stability and balance performance. The tool has demonstrated robust reliability and validity, and was considered appropriate for assessing mobility in older adults with cardiovascular disease in this study [30].

### 2.7 Statistical Analysis

All statistical analyses were performed using SPSS Version 24.0 (IBM Corp., Armonk, NY, United States), and visualizations were generated with GraphPad Prism version 10.0 (GraphPad Software, San Diego, CA, United States). The Kolmogorov–Smirnov test was applied to assess the normality of continuous variables. Normally distributed data were expressed as mean ± standard deviation (*x̄* ± *s*) and compared using independent‐sample *t* tests. Nonnormally distributed variables were reported as median and interquartile range (*M* [*P*_25_, *P*_75_]) and analysed using the Mann–Whitney *U* test. Categorical variables were presented as counts and percentages and compared using the chi‐square (*χ* ^*2*^) test or Fisher's exact test as appropriate. Ordinal data were evaluated using the Wilcoxon rank‐sum test.

To account for multiple comparisons involving EMG indices across multiple muscles, the Benjamini–Hochberg procedure was employed to control the false discovery rate (FDR). All *p* values from two‐group comparisons were FDR‐adjusted, with statistical significance defined as an adjusted two‐tailed *p* < 0.05.

EMG indicators with FDR‐adjusted *p* < 0.10 were first entered into binary logistic regression models (forward stepwise method) to identify potential predictors of frailty. Those EMG features, along with clinical characteristics and secondary outcomes (e.g., gait speed and Tinetti POMA score) showing significant group differences, were subsequently entered into a multivariable binary logistic regression model (enter method) to identify independent predictors while controlling for confounders. Finally, receiver operating characteristic (ROC) curve analysis was conducted for predictors retained in the multivariable model to evaluate their discriminative performance, including area under the curve (AUC), sensitivity, specificity and optimal cutoff values.

## 3 Results

### 3.1 Comparison of Clinical Characteristics, 10‐m Walk Test Time and Tinetti POMA Scores

Compared with the nonfrail group, frail older adults were significantly older, more likely to be on polypharmacy, required longer to complete the 10‐m walk test, and demonstrated significantly lower Tinetti POMA scores (all *p* < 0.05). The corresponding walking speeds were 1.08 ± 0.13 m/s in the nonfrail group and 0.79 ± 0.19 m/s in the frail group, with an absolute between‐group difference of 0.29 m/s. The absolute between‐group difference in Tinetti POMA score was 6.08 points. No significant between‐group differences were observed in sex, body mass index, educational attainment, living status, type of cardiovascular disease, comorbid diabetes, history of musculoskeletal injury, smoking or alcohol use (all *p* > 0.05). Detailed results are presented in Table 1.

**TABLE 1:** Comparison of clinical characteristics, 10‐m walk test time and Tinetti POMA scores between frail and nonfrail older adults with cardiovascular disease.

Variable	Nonfrail group (*n* = 75)	Frail group (*n* = 75)	*χ* ^*2*^, *t*, or *Z*	*p*
Sex, *n* (%)			0.4286	0.5127
Male	42 (56.00%)	38 (50.67%)		
Female	33 (44.00%)	37 (49.33%)		
Age (years)	68.55 ± 6.09	76.04 ± 7.57	6.681	< 0.0001*
Body Mass Index (kg/m^2^)	24.58 ± 3.28	24.78 ± 4.17	0.3169	0.7517
Educational level, *n* (%)			—	0.7969
Middle school or below	60 (80.00%)	61 (81.33%)		
High school/vocational	4 (5.33%)	2 (2.67%)		
College/university	11 (14.67%)	12 (16.00%)		
Living arrangement, *n* (%)			—	> 0.9999
Living alone	0 (0.00%)	1 (1.33%)		
Living with others	75 (100.00%)	74 (98.67%)		
Type of CVD, *n* (%)			—	0.7482
Coronary artery disease	35 (46.67%)	29 (38.67%)		
Heart failure	13 (17.33%)	19 (25.33%)		
Arrhythmia	14 (18.67%)	13 (17.33%)		
Cardiomyopathy	7 (9.33%)	5 (6.67%)		
Hypertension	3 (4.00%)	5 (6.67%)		
Valvular disease	3 (4.00%)	4 (5.33%)		
Polypharmacy, *n* (%)			13.05	0.0003*
Yes	53 (70.67%)	70 (93.33%)		
No	22 (29.33%)	5 (6.67%)		
Diabetes mellitus, *n* (%)			1.556	0.2123
Yes	19 (25.33%)	26 (34.67%)		
No	56 (74.67%)	49 (65.33%)		
History of musculoskeletal injury, *n* (%)			0.2308	0.6310
Yes	11 (14.67%)	9 (12.00%)		
No	64 (85.33%)	66 (88.00%)		
Smoking, *n* (%)			0.03885	0.8437
Yes	17 (22.67%)	16 (21.33%)		
No	58 (77.33%)	59 (78.67%)		
Alcohol consumption, *n* (%)			1.119	0.2900
Yes	6 (8.00%)	10 (13.33%)		
No	69 (92.00%)	65 (86.67%)		
10‐m walk time (s)	9.44 ± 1.21	13.34 ± 3.23	9.764	< 0.0001*
Tinetti POMA score	27.59 ± 0.92	21.51 ± 4.27	12.05	< 0.0001*

* Statistically significant at *p* < 0.05.

### 3.2 Comparison of Lower Limb Surface Electromyographic Parameters Between Frail and Nonfrail Groups

Between‐group comparisons of lower limb sEMG parameters revealed no statistically significant differences in AEMG, RMS, SI or muscle contribution ratio (Tables S1–S4). Among time‐domain variables, the frail group showed higher IEMG of the left semitendinosus than the nonfrail group (FDR‐adjusted *p* < 0.05; Figure 1A), whereas no other IEMG comparisons remained significant after FDR correction (Table S5). For muscle activation‐sequence rank, the frail group had a later activation order of the left rectus femoris than the nonfrail group (FDR‐adjusted *p* < 0.05; Figure 1B). No other muscle activation‐sequence ranks reached FDR‐adjusted statistical significance (Table S6).

**FIGURE 1:**
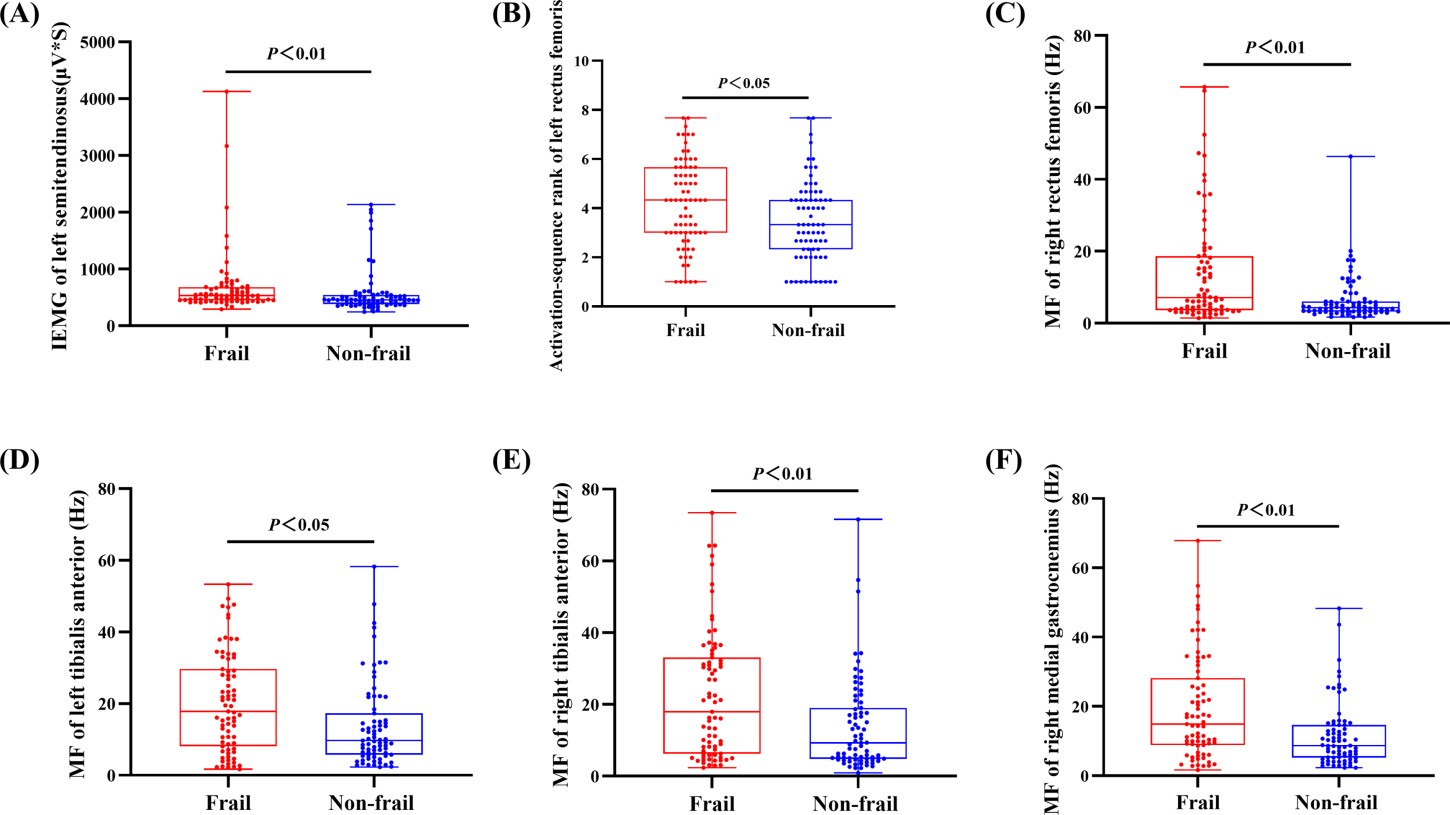
Box plots comparing lower limb surface electromyographic (sEMG) parameters between frail and nonfrail older adults. (A) IEMG of left semitendinosus; (B) Activation‐sequence rank of left rectus femoris; (C) MF of right rectus femoris; (D) MF of left tibialis anterior; (E) MF of right tibialis anterior; and (F) MF of right medial gastrocnemius.

In the frequency domain, frail participants showed significantly higher median MF values in the right rectus femoris, left tibialis anterior, right tibialis anterior and right medial gastrocnemius (all FDR‐adjusted *p* < 0.05; Figure 1C–F), whereas no between‐group differences were found in MF values for other muscles (Table S7). Similarly, MPF values were significantly elevated in the frail group for the left rectus femoris, right rectus femoris, left semitendinosus, left tibialis anterior, right tibialis anterior and right medial gastrocnemius (all FDR‐adjusted *p* < 0.05; Figure 2A–F), with no significant differences noted in other muscles (Table S8).

**FIGURE 2:**
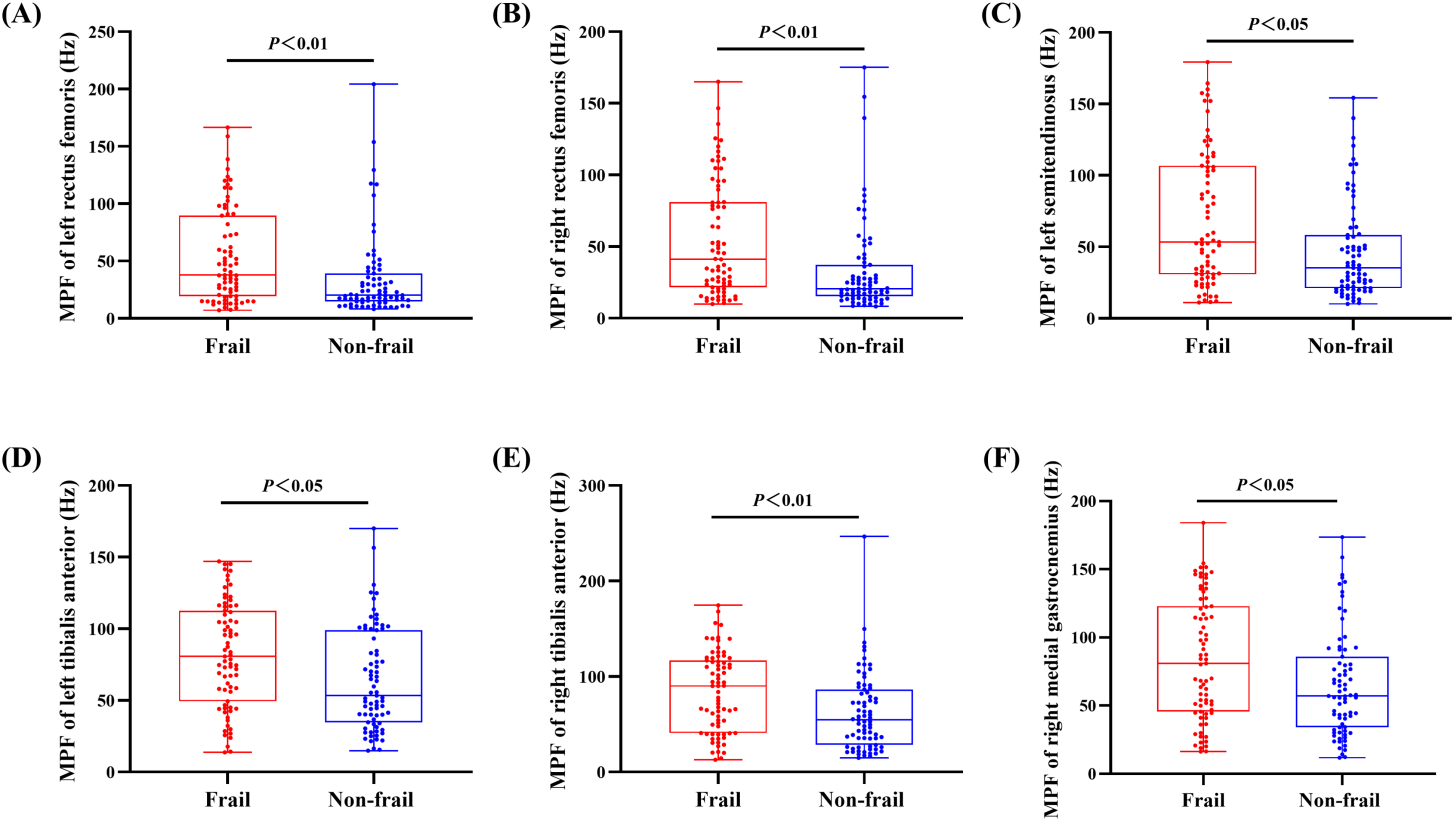
Box plots comparing lower limb surface electromyographic (sEMG) parameters between frail and nonfrail older adults. (A) MPF of left rectus femoris; (B) MPF of right rectus femoris; (C) MPF of left semitendinosus; (D) MPF of left tibialis anterior; (E) MPF of right tibialis anterior; and (F) MPF of right medial gastrocnemius.

### 3.3 Binary Logistic Regression Analysis of Factors Associated With Frailty in Older Adults With Cardiovascular Disease

Binary logistic regression was conducted with frailty status (frail vs. nonfrail) as the dependent variable. Surface electromyographic indicators with FDR‐adjusted *p* values < 0.10 in the between‐group comparisons were first entered into a forward stepwise regression to identify candidate predictors (detailed results are provided in Table S9). Three features were retained as significant predictors: MF of the right rectus femoris (OR = 1.064, 95% CI: 1.001–1.132, *p* = 0.045), MF of the right medial gastrocnemius (OR = 1.053, 95% CI: 1.008–1.101, *p* = 0.021) and activation‐sequence rank of the right tibialis anterior (OR = 0.643, 95% CI: 0.455–0.908, *p* = 0.012).

These three sEMG parameters, together with age, polypharmacy status, 10‐m walk test time and Tinetti POMA score, were subsequently included in a multivariable logistic regression model using the enter method to control for potential confounding factors and identify factors independently associated with frailty. In this model, MF of the right rectus femoris (OR = 1.067, 95% CI: 1.003–1.135, *p* = 0.038), MF of the right medial gastrocnemius (OR = 1.054, 95% CI: 1.009–1.102, *p* = 0.019), activation‐sequence rank of the right tibialis anterior (OR = 0.640, 95% CI: 0.448–0.914, *p* = 0.014), age (OR = 1.128, 95% CI: 1.044–1.218, *p* = 0.002) and Tinetti POMA score (OR = 0.784, 95% CI: 0.669–0.918, *p* = 0.003) remained independently associated with frailty (Figure 3). Higher MF values of the right rectus femoris and right medial gastrocnemius, as well as older age, were independently associated with higher odds of frailty, whereas higher Tinetti POMA scores and higher activation‐sequence ranks of the right tibialis anterior were independently associated with lower odds of frailty.

**FIGURE 3:**
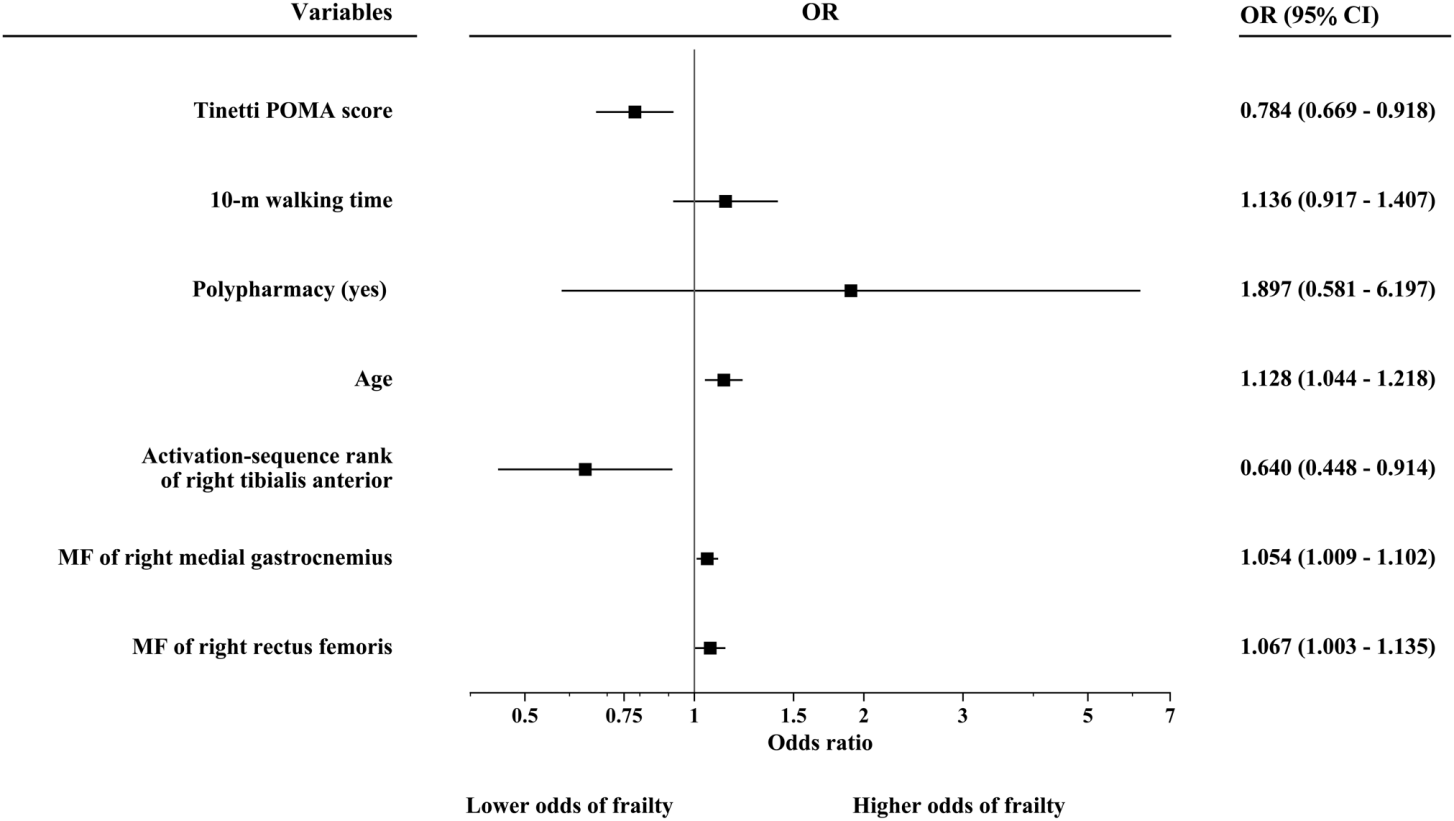
Forest plot of multivariable logistic regression analysis of factors associated with frailty in older adults with cardiovascular disease. The vertical reference line indicates OR = 1. Values to the right of the reference line indicate higher odds of frailty, whereas values to the left indicate lower odds of frailty.

The Hosmer–Lemeshow goodness‐of‐fit tests demonstrated acceptable model fit for both the forward stepwise model (*χ* ^*2*^ = 9.058, df = 8, *p* = 0.337) and the multivariable enter model (*χ* ^*2*^ = 9.997, df = 8, *p* = 0.265), indicating no significant deviation between predicted and observed outcomes. Variance inflation factor (VIF) diagnostics revealed no evidence of multicollinearity, with all VIFs < 5.

### 3.4 ROC Curve Analysis for Frailty Identification in Older Adults With Cardiovascular Disease

Receiver operating characteristic (ROC) curve analysis was performed to assess the discriminative ability of individual and combined indicators for frailty identification in older adults with CVD. The area under the curve (AUC) values for MF of the right rectus femoris, MF of the right medial gastrocnemius, activation‐sequence rank of the right tibialis anterior, age and Tinetti POMA score were 0.6563, 0.6591, 0.6060, 0.7692 and 0.7314, respectively. The corresponding sensitivities were 0.7467, 0.8400, 0.4800, 0.8267 and 0.7067, whereas specificities were 0.5867, 0.4800, 0.7067, 0.5467 and 0.6800.

When combined, the three retained sEMG features yielded an AUC of 0.7387 (sensitivity = 0.8400, specificity = 0.5333). Adding age increased the AUC to 0.8418 (sensitivity = 0.8933, specificity = 0.6400). Incorporating both age and Tinetti POMA score further improved the model performance, with an AUC of 0.8740, sensitivity of 0.9200 and specificity of 0.7067 (Figure 4; Table 2).

**FIGURE 4:**
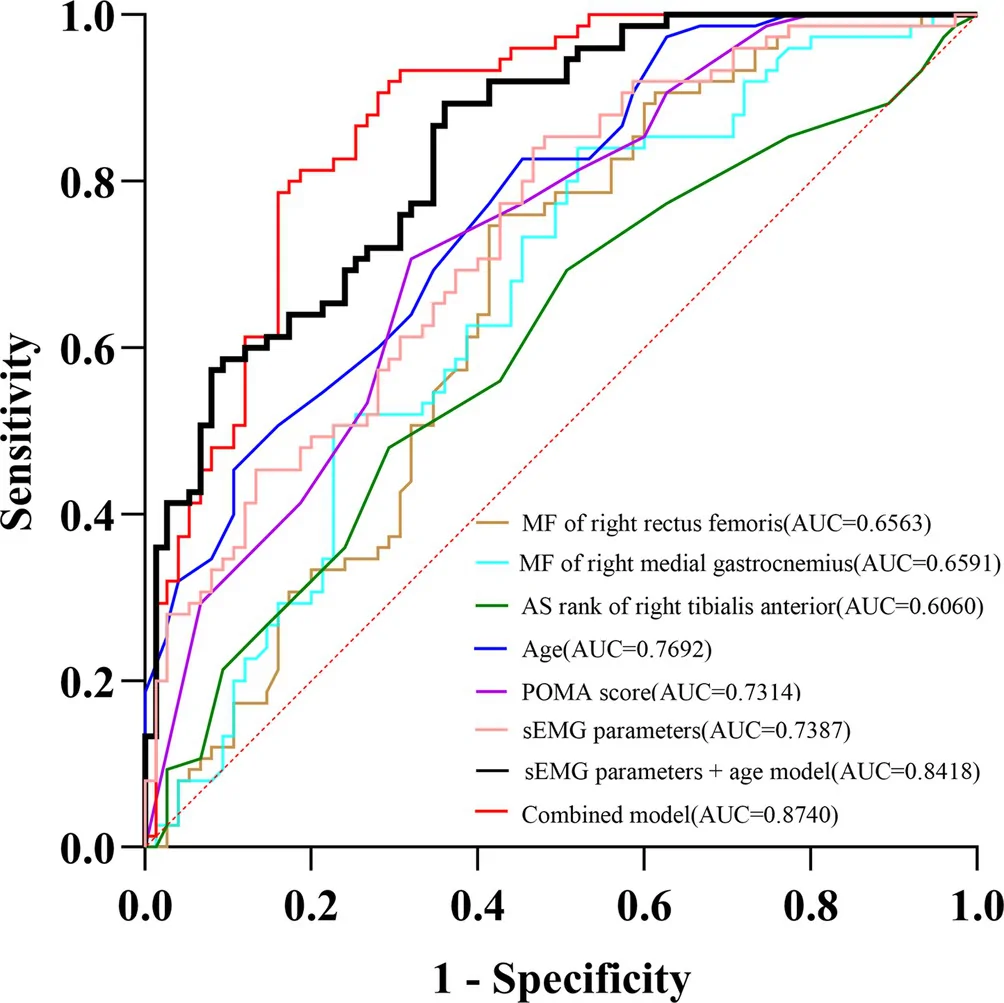
ROC curves for frailty identification in older adults with cardiovascular disease.

**TABLE 2:** ROC curve analysis for frailty identification in older adults with cardiovascular disease.

Variable	AUC	95% CI	*p*	Optimal cutoff	Sensitivity	Specificity	Youden's Index
MF of right rectus femoris	0.6563	0.5679~0.7447	0.0010	6.005	0.7467	0.5867	0.3334
MF of right medial gastrocnemius	0.6591	0.5714~0.7468	0.0008	16.41	0.8400	0.4800	0.3200
Activation‐sequence rank of right tibialis anterior	0.6060	0.5154~0.6965	0.0251	5.167	0.4800	0.7067	0.1867
Age	0.7692	0.6961~0.8424	< 0.0001	74.50	0.8267	0.5467	0.3734
POMA score	0.7314	0.6521~0.8107	< 0.0001	24.50	0.7067	0.6800	0.3867
sEMG parameters	0.7387	0.6606~0.8167	< 0.0001	—	0.8400	0.5333	0.3733
sEMG parameters + age model	0.8418	0.7812~0.9024	< 0.0001	—	0.8933	0.6400	0.5333
Combined model	0.8740	0.8178~0.9302	< 0.0001	—	0.9200	0.7067	0.6267

## 4 Discussion

Frailty has emerged as an essential ‘additional vital sign’ in the care and research of older adults with cardiovascular disease (CVD). Frailty and CVD share common pathophysiological mechanisms, including chronic inflammation [S1], oxidative stress [S2] and insulin resistance [S3], which may mutually exacerbate each other. Frailty not only serves as a predictor of adverse outcomes in patients with CVD but may also be precipitated or worsened by the presence of cardiovascular pathology [S4]. Increasing evidence suggests that frailty could potentially be recognized as an independent risk factor for CVD in the future [S3, S5].

As a dynamic and potentially reversible syndrome, frailty requires timely identification, especially among older adults with chronic CVD, to enable early and personalized intervention. However, no universally endorsed standard currently exists for frailty screening [31]. Tools such as the Fried frailty phenotype and the Frailty Index by Rockwood et al. [28], [S6] are widely used but often entail complex administration, rely heavily on subjective clinical judgement and lack objective biological correlates. An optimal frailty assessment instrument should be simple, quantitatively robust, objectively validated and generalizable across populations, producing consistent and reproducible results [32]. In this context, the search for feasible and reliable biomarkers of frailty remains a pressing priority.

Given the close association between skeletal muscle degeneration, neuromuscular electrophysiology and frailty [S1], [15], this study examined surface electromyographic (sEMG) characteristics of key lower limb muscles in frail versus nonfrail older adults with CVD. Compared with their nonfrail counterparts, frail individuals were older, more frequently exposed to polypharmacy, exhibited longer 10‐m walk test times and scored lower on the Tinetti POMA, indicating poorer functional mobility consistent with clinical frailty. In particular, the mobility and balance measures provided functional support for the Fried phenotype‐based group separation. Frail participants walked 0.29 m/s slower than nonfrail participants, a difference exceeding commonly cited thresholds for meaningful change in gait speed among older adults [S7]. They also had a 6.08‐point lower Tinetti POMA score, indicating poorer gait and balance performance; the Tinetti POMA is widely used to assess mobility limitations and fall risk in older adults, with recent evidence supporting its reliability and validity in Chinese older adults [S8, S9]. Therefore, the Fried phenotype‐based group separation in this study was accompanied by functionally meaningful differences in mobility, gait stability and balance performance. Notably, the frail group showed significantly elevated integrated EMG (IEMG) in the left semitendinosus, a later activation‐sequence rank of the left rectus femoris and higher median frequency (MF) in the right rectus femoris, bilateral tibialis anterior and right medial gastrocnemius. Mean power frequency (MPF) was also increased in six muscles—bilateral rectus femoris, left semitendinosus, bilateral tibialis anterior and right medial gastrocnemius—suggesting a shift in neuromuscular activation patterns.

These changes in neuromuscular electrical activity are closely linked to degenerative alterations in muscle tissue structure. Frail older adults often exhibit reduced muscle fibre count and decreased cross‐sectional area (CSA), particularly involving Type II (fast‐twitch) fibres, which are prone to atrophy, selective loss and phenotypic transformation towards Type I (slow‐twitch) fibres [33, 34], [S10]. Because Type II fibres are responsible for rapid, high‐intensity contractions, their deterioration compromises muscular power and dynamic stability, potentially leading to delayed initiation of functional movements and diminished neural drive efficiency. Such structural deterioration is reflected in sEMG signals as elevated MF and MPF, indicating compensatory recruitment of additional motor units via increased firing rates. The widespread increases in MPF and MF observed in the frail group in this study likely represent the electrophysiological manifestations of underlying pathological muscle fibre remodelling.

The findings of this study suggest that frail individuals may require greater muscle contraction or activation to perform the same motor tasks and exhibit delayed initiation of movements, which corresponds with their prolonged walking times. To compensate for reduced muscle strength, these individuals may recruit a greater number of fast‐twitch muscle fibres, which are characterized by higher discharge frequencies. Moreover, the muscle activation patterns in the frail group appear to be altered, showing a tendency towards more explosive or phasic activation strategies. This observation is consistent with prior studies. For example, in a study examining daily muscle activity, frail women showed a 73% reduction in upper limb muscle inactivity periods (gaps) and a 48% increase in burst duration compared to nonfrail women, indicating a more continuous and forceful activation pattern [35]. Similarly, Jones et al. [36] reported that frail individuals exhibited a higher frequency of EMG bursts and fewer gaps during the performance of specific functional tasks, further supporting the notion of a compensatory neuromuscular adaptation in the frail phenotype.

Such adaptations may reflect an altered central motor control strategy that compensates for impaired conduction and diminished contractile capacity through high‐frequency, energetically costly activation patterns. Although these adaptations may temporarily preserve mobility, they are likely to increase fatigue and instability, elevating the risk of falls and further decline. From a physiological perspective, elevated MF and MPF reflect increased motor unit discharge rates, denoting a ‘high neural cost’ strategy to counteract strength deficits. These findings support the concept of ‘overactivation–inefficient mobilization’ as a hallmark of frailty and underscore the potential utility of sEMG‐derived parameters as objective, quantifiable biomarkers for frailty identification and progression monitoring.

Multivariable logistic regression analysis identified MF of the right rectus femoris, MF of the right medial gastrocnemius, activation‐sequence rank of the right tibialis anterior, age and Tinetti POMA score as factors independently associated with frailty. Higher MF values of the right rectus femoris and right medial gastrocnemius were associated with higher odds of frailty after adjustment, whereas higher activation‐sequence rank of the right tibialis anterior was inversely associated with frailty. Advancing age and lower Tinetti POMA scores were also independently associated with frailty. Receiver operating characteristic (ROC) curve analysis further demonstrated that, although individual sEMG variables offered limited predictive utility, combining three sEMG features substantially improved model performance. The ‘sEMG + age’ model, independent of performance‐based clinical scales, achieved strong discriminatory power (AUC = 0.8418; sensitivity = 0.8933; specificity = 0.6400), approaching that of the full model incorporating Tinetti POMA scores (AUC = 0.8740). This finding suggests that sEMG features, particularly when combined with age, may offer a practical balance between feasibility and predictive performance. Therefore, lower limb sEMG features may serve as candidate objective markers that complement conventional functional assessments for frailty identification.

Notably, all sEMG parameters retained in the multivariable model were derived from the right (dominant) limb, suggesting that the dominant side, which is frequently engaged in daily functional activities, may be sensitive to frailty‐related neuromuscular alterations during walking. However, the influence of limb dominance on gait symmetry and lower limb neuromuscular control remains complex and requires further investigation [S11]. Previous research has demonstrated that short‐duration upper limb EMG signals captured during functional tasks can accurately classify frailty status in older adults, with classification accuracy approaching 90% [36]. The present study extends this evidence to dynamic lower limb walking tasks, supporting the potential of sEMG‐derived features as candidate objective markers for frailty assessment. The abnormal frequency‐domain features observed in the right rectus femoris and gastrocnemius muscles—key muscles involved in weight‐bearing and propulsion—may reflect neuromuscular fatigue or compensatory adaptations. In contrast, the inverse association between activation‐sequence rank of the right tibialis anterior and frailty should be interpreted cautiously. Activation‐sequence rank in this study was a unitless ordinal measure of relative onset order among the recorded muscles rather than an absolute measure of recruitment timing. Given the role of the tibialis anterior in ankle dorsiflexion and foot clearance during walking [S12], this finding may reflect altered intermuscle coordination or compensatory reorganization during gait. Further studies using absolute onset timing, gait‐cycle‐normalized activation timing and external validation are needed to clarify its physiological and clinical significance.

Objective assessment of frailty remains in the exploratory stage. Surface electromyography, as a bioelectrical signal reflecting neuromuscular function, is capable of detecting early abnormalities preceding overt functional decline. Compared with conventional frailty assessment tools such as the Fried phenotype, Clinical Frailty Scale (CFS) and Frailty Index (FI), the ‘sEMG + age’ model developed in this study offers greater objectivity and potential for automation, suggesting its potential for rapid frailty identification in cardiovascular populations. Conventional frailty scales remain clinically useful, but some require physical performance testing, clinical judgement or multidomain data collection, which may limit their rapid implementation in some patients with CVD. For instance, grip strength testing poses potential risks in individuals with heart failure [37, 38]. In contrast, sEMG provides a noninvasive, dynamic and quantifiable measure that is particularly advantageous for detecting neuromuscular alterations associated with frailty in patients with preserved physical function. Importantly, this model aligns with the emerging consensus on the ideal characteristics of frailty assessment tools—namely, sensitivity, specificity, brevity (< 5 min), reproducibility and cardiovascular‐specific validation [39], [S13]. Beyond identifying key sEMG features associated with frailty in older adults with CVD, this study proposes a potential framework for objective frailty assessment and provides a conceptual and methodological foundation for the development of novel, objective and automated frailty assessment tools. With the increasing accessibility of portable sEMG devices, this model holds promise for integration into wearable technologies or remote health monitoring platforms, supporting future dynamic monitoring and individualized intervention planning in older adults with CVD.

Despite the study's innovation and practical implications, several limitations should be acknowledged. First, the sample was relatively small and drawn from a single centre, which may limit generalizability. Second, the case–control design precludes causal inference between EMG parameters and frailty. Third, although major confounders were adjusted for, unmeasured confounding cannot be excluded. Fourth, MVC normalization was not performed for time‐domain sEMG parameters because maximal voluntary contraction testing may be difficult to perform safely and consistently in frail older adults with cardiovascular disease. Despite the standardized 10‐m walk test and identical signal‐processing pipeline, AEMG, RMS and IEMG may be influenced by interindividual differences in subcutaneous tissue, electrode‐skin impedance and electrode placement; therefore, they should be interpreted as walking task‐derived sEMG measures rather than %MVC‐normalized activation levels. Fifth, the model did not include relevant domains such as cognition, nutrition and inflammation, which may have affected predictive performance. Finally, EMG data were collected at a single time point during a 10‐m walk, without longitudinal measurements. Future multicentre, prospective studies with serial sEMG assessments are needed to confirm the value of lower limb sEMG features as candidate markers for frailty identification and monitoring.

## 5 Conclusions

In conclusion, this study identified distinct lower limb surface electromyographic (sEMG) features that differentiate frail from nonfrail older adults with cardiovascular disease. Notably, the sEMG variables independently associated with frailty included median frequency of the right rectus femoris, median frequency of the right medial gastrocnemius and activation‐sequence rank of the right tibialis anterior. Among the tested models, the ‘sEMG + age’ model demonstrated good predictive performance without relying on performance‐based clinical scales, offering a practical balance between sensitivity, simplicity and potential automation. These findings suggest that lower limb sEMG‐derived features may serve as candidate objective markers for frailty identification in older adults with CVD and may support future applications in early risk detection, dynamic monitoring and individualized intervention planning.

## Funding

This work was supported by the Chinese Nursing Association (Grant no. ZHKY202313). The funder had no role in study design, data collection and analysis, decision to publish or preparation of the manuscript.

## Conflicts of Interest

The authors declare no conflicts of interest.

## Supporting information


**Table S1:** Comparison of lower‐limb AEMG between frail and non‐frail older adults [M (P25, P75)].
**Table S2:** Comparison of lower‐limb RMS between frail and non‐frail older adults [M (P25, P75)].
**Table S3:** Comparison of symmetry index in lower‐limb muscles between frail and non‐frail older adults [M (P25, P75)].
**Table S4:** Comparison of lower‐limb muscle contribution ratio between frail and non‐frail older adults [M (P25, P75)].
**Table S5:** Comparison of lower‐limb IEMG between frail and non‐frail older adults [M (P25, P75)].
**Table S6:** Comparison of lower‐limb muscle activation‐sequence rank between frail and non‐frail older adults [M (P25, P75)].
**Table S7:** Comparison of lower‐limb MF between frail and non‐frail older adults [M (P25, P75)].
**Table S8:** Comparison of lower‐limb MPF between frail and non‐frail older adults [M (P25, P75)].
**Table S9:** Forward stepwise binary logistic regression analysis for candidate sEMG predictors of frailty in older adults with cardiovascular disease.

**Figure S1:** Electrode placement and stabilization for surface electromyography (sEMG) in lower‐limb muscles.

**Figure S2:** Representative raw surface electromyography waveforms during walking.
