# Supplementary material for: Surface Electromyographic Characteristics of Lower Limb Muscles in Frail Older Adults With Cardiovascular Disease: A Case–Control Study
Source: J Cachexia Sarcopenia Muscle. 2026 Oct 5;17(5):e70387. doi: 10.1002/jcsm.70387 (PMC13635801; doi:10.1002/jcsm.70387)
Supplement: Supplementary file 1 [file JCSM-17-e70387-s003.docx]

**Surface electromyographic characteristics of lower limb muscles in frail older adults with cardiovascular disease: a case-control study**

**Supplementary Online Content**

**Supplementary Table 1. Comparison of lower-limb AEMG between frail and non-frail older adults [M (P25, P75)]**

**Supplementary Table 2. Comparison of lower-limb RMS between frail and non-frail older adults [M (P25, P75)]**

**Supplementary Table 3. Comparison of symmetry index in lower-limb muscles between frail and non-frail older adults [M (P25, P75)]**

**Supplementary Table 4. Comparison of lower-limb muscle contribution ratio between frail and non-frail older adults [M (P25, P75)]**

**Supplementary Table 5. Comparison of lower-limb IEMG between frail and non-frail older adults [M (P25, P75)]**

**Supplementary Table 6. Comparison of lower-limb muscle activation-sequence rank between frail and non-frail older adults [M (P25, P75)]**

**Supplementary Table 7. Comparison of lower-limb MF between frail and non-frail older adults [M (P25, P75)]**

**Supplementary Table 8. Comparison of lower-limb MPF between frail and non-frail older adults [M (P25, P75)]**

**Supplementary Table 9. Forward stepwise binary logistic regression analysis for candidate sEMG predictors of frailty in older adults with cardiovascular disease**

**Supplementary Figure 1. Electrode placement and stabilization for surface electromyography (sEMG) in lower-limb muscles.**

**Supplementary Figure 2. Representative raw surface electromyography waveforms during walking**

**Supplementary References**

**Supplementary Table 1. Comparison of lower-limb AEMG between frail and non-frail older adults [M (P25, P75)]**

**Unit: μV**

| **Muscle** | **Non-frail group (n=75)** | **Frail group (n=75)** | ***Z*** | ***P*** | **FDR *P*** |
| --- | --- | --- | --- | --- | --- |
| **Rectus femoris (left)** | 92.22 (83.38, 109.3) | 92.86 (81.15, 104.4) | -0.72 | 0.4722 | 0.7256 |
| **Rectus femoris (right)** | 94.02 (84.52, 100.5) | 91.39 (79.99, 104.7) | -1.04 | 0.3022 | 0.5849 |
| **Semitendinosus (left)** | 91.68 (80.12, 102.1) | 91.92 (84.44, 109.5) | -1.18 | 0.2390 | 0.4945 |
| **Semitendinosus (right)** | 91.50 (84.16, 100.8) | 90.38 (79.07, 102.8) | -0.52 | 0.6076 | 0.7595 |
| **Tibialis anterior (left)** | 94.56 (83.24, 104.9) | 94.62 (85.63, 101.9) | -0.17 | 0.8668 | 0.9043 |
| **Tibialis anterior (right)** | 94.82 (84.02, 104.7) | 92.15 (81.65, 102.2) | -0.93 | 0.3523 | 0.6217 |
| **Medial gastrocnemius (left)** | 94.31 (85.10, 111.3) | 96.63 (84.55, 105.6) | -0.21 | 0.8367 | 0.9043 |
| **Medial gastrocnemius (right)** | 96.95 (90.06, 109.5) | 94.89 (81.36, 108.6) | -1.34 | 0.1799 | 0.4151 |

**Supplementary Table 2. Comparison of lower-limb RMS between frail and non-frail older adults [M (P25, P75)]**

**Unit: μV**

| **Muscle** | **Non-frail group (n=75)** | **Frail group**  **(n=75)** | ***Z*** | ***P*** | **FDR *P*** |
| --- | --- | --- | --- | --- | --- |
| **Rectus femoris (left)** | 56.12 (47.29, 70.22) | 56.88 (45.22, 70.18) | -0.09 | 0.9253 | 0.9505 |
| **Rectus femoris (right)** | 55.87 (47.03, 67.61) | 54.14 (41.82, 67.30) | -0.81 | 0.4156 | 0.6234 |
| **Semitendinosus (left)** | 52.32 (45.16, 63.04) | 54.47 (44.70, 66.73) | -0.25 | 0.8053 | 0.9332 |
| **Semitendinosus (right)** | 53.30 (45.56, 62.74) | 51.34 (41.82, 64.89) | -0.19 | 0.8517 | 0.9415 |
| **Tibialis anterior (left)** | 53.31 (45.80, 64.22) | 54.83 (44.91, 65.42) | -0.02 | 0.9862 | 0.9862 |
| **Tibialis anterior (right)** | 53.37 (46.61, 63.65) | 55.39 (42.87, 66.23) | -0.12 | 0.9020 | 0.9505 |
| **Medial gastrocnemius (left)** | 52.93 (45.84, 63.16) | 54.66 (47.26, 66.48) | -0.59 | 0.5576 | 0.7256 |
| **Medial gastrocnemius (right)** | 55.47 (46.39, 63.89) | 56.01 (46.36, 67.62) | -0.18 | 0.8546 | 0.9415 |

**Supplementary Table 3. Comparison of symmetry index in lower-limb muscles between frail and non-frail older adults [M (P25, P75)]**

| **Muscle** | **Non-frail group (n = 75)** | **Frail group (n = 75)** | ***Z*** | ***P*** | **FDR *P*** |
| --- | --- | --- | --- | --- | --- |
| **Rectus femoris** | 0.000 (-0.090, 0.120) | 0.000 (-0.180, 0.080) | -0.70 | 0.4837 | 0.7256 |
| **Semitendinosus** | 0.000 (-0.09000, 0.09000) | 0.02000 (-0.09000, 0.1500) | 0.86 | 0.3918 | 0.6530 |
| **Tibialis anterior** | 0.010 (-0.100, 0.090) | 0.020 (-0.070, 0.110) | 0.46 | 0.6426 | 0.7711 |
| **Medial gastrocnemius** | 0.01000 (-0.09000, 0.09000) | 0.01000 (-0.1000, 0.1000) | -0.058 | 0.9544 | 0.9544 |

**Supplementary Table 4. Comparison of lower-limb muscle contribution ratio between frail and non-frail older adults [M (P25, P75)]**

**Unit: %**

| **Muscle** | **Non-frail group (n=75)** | **Frail group (n=75)** | ***Z*** | ***P*** | **FDR *P*** |
| --- | --- | --- | --- | --- | --- |
| **Rectus femoris (left)** | 11.87 (10.08, 13.95) | 11.89 (9.850, 13.86) | 0.1584 | 0.8742 | 0.9043 |
| **Rectus femoris (right)** | 12.02 (10.22, 13.51) | 11.63 (10.28, 13.32) | 0.764 | 0.4471 | 0.7250 |
| **Semitendinosus (left)** | 10.91 (9.260, 13.20) | 12.15 (9.980, 14.13) | -2.0554 | 0.0391 | 0.1303 |
| **Semitendinosus (right)** | 11.44 (9.880, 12.96) | 11.29 (9.980, 13.31) | -0.18 | 0.8580 | 0.9043 |
| **Tibialis anterior (left)** | 11.39 (9.990, 13.93) | 12.50 (9.990, 13.95) | -0.49 | 0.6241 | 0.7642 |
| **Tibialis anterior (right)** | 12.19 (10.13, 13.57) | 11.58 (10.44, 13.16) | -0.491 | 0.5307 | 0.7400 |
| **Medial gastrocnemius (left)** | 12.04 (10.47, 13.91) | 12.07 (10.67, 13.90) | -0.115 | 0.9097 | 0.9251 |
| **Medial gastrocnemius (right)** | 12.64 (10.86, 14.01) | 11.82 (10.36, 14.04) | -0.63 | 0.5270 | 0.7400 |

**Supplementary Table 5. Comparison of lower-limb IEMG between frail and non-frail older adults [M (P25, P75)]**

**Unit: μV*S**

| **Muscle** | **Non-Frail Group (n = 75)** | **Frail Group**  **(n = 75)** | ***Z*** | ***P*** | **FDR *P*** |
| --- | --- | --- | --- | --- | --- |
| **Rectus femoris (left)** | 468.8 (409.4, 637.0) | 516.2 (448.1, 626.3) | -1.55 | 0.1226 | 0.3198 |
| **Rectus femoris (right)** | 480.5 (421.4, 567.5) | 515.0 (430.8, 695.2) | -1.24 | 0.2169 | 0.4820 |
| **Semitendinosus (left)** | 455.5 (384.5, 542.0) | 534.4 (451.3, 680.7) | -3.42 | 0.0006 | 0.0084* |
| **Semitendinosus (right)** | 459.1 (410.3, 546.4) | 516.5 (437.4, 636.5) | -2.24 | 0.0253 | 0.0935 |
| **Tibialis anterior (left)** | 481.9 (411.1, 561.6) | 525.5 (447.4, 624.4) | -2.22 | 0.0265 | 0.0935 |
| **Tibialis anterior (right)** | 475.5 (409.4, 555.4) | 514.5 (431.3, 598.8) | -1.81 | 0.0698 | 0.1994 |
| **Medial gastrocnemius (left)** | 501.3 (425.5, 611.1) | 528.1 (449.7, 657.4) | -1.14 | 0.2571 | 0.5142 |
| **Medial gastrocnemius (right)** | 497.0 (432.9, 602.1) | 541.6 (432.7, 694.7) | -0.95 | 0.3426 | 0.6217 |

**Supplementary Table 6. Comparison of lower-limb muscle activation-sequence rank between frail and non-frail older adults [M (P25, P75)]**

| **Muscle** | **Non-frail Group**  **(n = 75)** | **Frail Group**  **(n = 75)** | ***Z*** | ***P*** | **FDR *P*** |
| --- | --- | --- | --- | --- | --- |
| **Rectus femoris (left)** | 3.333 (2.333, 4.333) | 4.333 (3.000, 5.667) | -2.695 | 0.0074 | 0.0404* |
| **Rectus femoris (right)** | 3.000 (2.333, 4.667) | 4.000 (2.667, 5.333) | -1.439 | 0.1503 | 0.3758 |
| **Semitendinosus (left)** | 4.667 (3.333, 5.333) | 4.000 (3.000, 5.667) | -0.730 | 0.4654 | 0.7256 |
| **Semitendinosus (right)** | 4.667 (3.333, 5.667) | 4.333 (3.333, 5.333) | 1.010 | 0.3139 | 0.5886 |
| **Tibialis anterior (left)** | 4.670 (3.670, 5.670) | 4.670 (3.670, 5.330) | -0.290 | 0.7702 | 0.9043 |
| **Tibialis anterior (right)** | 5.000 (4.333, 6.000) | 4.667 (4.000, 5.333) | -2.240 | 0.0243 | 0.0935 |
| **Medial gastrocnemius (left)** | 5.000 (4.000, 6.333) | 5.000 (4.000, 5.667) | -0.586 | 0.5581 | 0.7400 |
| **Medial gastrocnemius (right)** | 5.333 (4.333, 6.000) | 5.000 (4.667, 5.667) | -0.562 | 0.5739 | 0.7400 |

**Supplementary Table 7. Comparison of lower-limb MF between frail and non-frail older adults [M (P25, P75)]**

**Unit: Hz**

| **Muscle** | **Non-Frail Group (n = 75)** | **Frail Group (n = 75)** | ***Z*** | ***P*** | **FDR *P*** |
| --- | --- | --- | --- | --- | --- |
| **Rectus femoris (left)** | 4.333 (2.780, 5.823) | 5.400 (3.400, 14.10) | -2.35 | 0.0185 | 0.0793 |
| **Rectus femoris (right)** | 4.323 (3.233, 6.043) | 7.067 (3.617, 18.64) | 3.25 | 0.0009 | 0.0086* |
| **Semitendinosus (left)** | 4.717 (3.430, 8.750) | 9.080 (3.577, 26.14) | -2.415 | 0.0155 | 0.0715 |
| **Semitendinosus (right)** | 6.333 (3.733, 12.07) | 8.467 (3.733, 26.43) | -1.867 | 0.0610 | 0.1926 |
| **Tibialis anterior (left)** | 9.667 (5.790, 17.29) | 17.80 (8.067, 29.60) | 3.036 | 0.0024 | 0.0144* |
| **Tibialis anterior (right)** | 9.267 (4.817, 19.03) | 17.95 (6.310, 33.11) | 3.27 | 0.0010 | 0.0086* |
| **Medial gastrocnemius (left)** | 10.50 (6.000, 14.61) | 12.27 (5.267, 29.25) | 1.20 | 0.2287 | 0.4901 |
| **Medial gastrocnemius (right)** | 8.667 (5.200, 14.60) | 14.88 (8.800, 28.20) | 3.38 | 0.0007 | 0.0084* |

**Supplementary Table 8. Comparison of lower-limb MPF between frail and non-frail older adults [M (P25, P75)]**

**Unit: Hz**

| **Muscle** | **Non-Frail Group (n = 75)** | **Frail Group (n = 75)** | ***Z*** | ***P*** | **FDR *P*** |
| --- | --- | --- | --- | --- | --- |
| **Rectus femoris (left)** | 20.41 (14.98, 39.33) | 37.91 (19.38, 89.69) | 3.44 | 0.0006 | 0.0084* |
| **Rectus femoris (right)** | 20.55 (15.18, 37.33) | 41.02 (21.61, 81.06) | 3.72 | <0.0001 | 0.0060* |
| **Semitendinosus (left)** | 35.17 (21.11, 58.24) | 53.46 (30.68, 106.7) | -3.13 | 0.0017 | 0.0128* |
| **Semitendinosus (right)** | 41.71 (25.72, 78.02) | 55.07 (25.87, 94.28) | 1.65 | 0.0983 | 0.2681 |
| **Tibialis anterior (left)** | 53.49 (34.79, 99.02) | 80.76 (49.44, 112.6) | 13.73 | 0.0024 | 0.0144* |
| **Tibialis anterior (right)** | 54.65 (28.41, 86.49) | 90.08 (40.84, 116.9) | 14.09 | 0.0007 | 0.0084* |
| **Medial gastrocnemius (left)** | 55.87 (37.33, 86.40) | 74.13 (33.31, 124.2) | 1.37 | 0.1729 | 0.4150 |
| **Medial gastrocnemius (right)** | 57.11 (34.07, 85.84) | 80.87 (45.59, 123.00) | -2.59 | 0.0094 | 0.0470* |

**Supplementary Table 9. Forward stepwise binary logistic regression analysis for candidate sEMG predictors of frailty in older adults with cardiovascular disease**

| **Step** | **Variable** | **B** | **SE** | **Wald χ²** | **df** | ***P*** | **OR** | **95% *CI* Lower** | **95% *CI* Upper** |
| --- | --- | --- | --- | --- | --- | --- | --- | --- | --- |
| **1** | MF of right rectus femoris | 0.077 | 0.023 | 10.885 | 1 | 0.001 | 1.08 | 1.032 | 1.131 |
| **2** | MF of right medial gastrocnemius | 0.037 | 0.017 | 4.851 | 1 | 0.028 | 1.038 | 1.004 | 1.073 |
|  | MF of right rectus femoris | 0.057 | 0.023 | 6.08 | 1 | 0.014 | 1.058 | 1.012 | 1.107 |
| **3** | MF of right medial gastrocnemius | 0.043 | 0.018 | 5.893 | 1 | 0.015 | 1.044 | 1.008 | 1.081 |
|  | MF of right rectus femoris | 0.061 | 0.024 | 6.608 | 1 | 0.010 | 1.063 | 1.015 | 1.114 |
|  | AS of right tibialis anterior | -0.359 | 0.15 | 5.732 | 1 | 0.017 | 0.699 | 0.521 | 0.937 |

**Hosmer–Lemeshow goodness-of-fit test: Step 1: χ² = 5.520, df = 8, P = 0.701; Step 2: χ² = 10.010, df = 8, P = 0.264; Step 3: χ² = 9.058, df = 8, P = 0.337.**

**Supplementary Figure 1. Electrode placement and stabilization for surface electromyography (sEMG) in lower-limb muscles. (A) Anterior view showing the placement of surface electrodes over the rectus femoris and tibialis anterior; (B) Posterior view showing the placement of surface electrodes over the semitendinosus and medial gastrocnemius; (C) Anterior view of electrode fixation with elastic bandages; (D) Posterior view of electrode fixation with elastic bandages.**

**Supplementary Figure 2. Representative raw surface electromyography waveforms during walking**

**Supplementary References**

S1. Kochlik B, Franz K, Henning T, et al. Frailty is characterized by biomarker patterns reflecting inflammation or muscle catabolism in multi-morbid patients. J Cachexia Sarcopenia Muscle. 2023;14(1):157-166.

S2. de Castro GS, Simoes E, Lima JDCC, et al. Human cachexia induces changes in mitochondria, autophagy and apoptosis in the skeletal muscle. Cancers 2019;11(9):1264.

S3. Boreskie KF, Oldfield CJ, Hay JL, et al. Myokines as biomarkers of frailty and cardiovascular disease risk in females. Exp Gerontol 2020;133:110859.

S4. Afilalo J. Frailty in patients with cardiovascular disease: why, when, and how to measure. Curr Cardiovasc Risk Rep 2011;5(5):467-472.

S5. Soto ME, Pérez-Torres I, Rubio-Ruiz ME, et al. Frailty and the interactions between skeletal muscle, bone, and adipose tissue-impact on cardiovascular disease and possible therapeutic measures. Int J Mol Sci 2023;24(5):4534.

S6. Rockwood K, Song X, MacKnight C, et al. A global clinical measure of fitness and frailty in elderly people. CMAJ 2005;173(5):489-495.

**S7. Perera S, Mody SH, Woodman RC, Studenski SA. Meaningful change and responsiveness in common physical performance measures in older adults. J Am Geriatr Soc. 2006;54(5):743-749.**

**S8. Omaña H, Bezaire K, Brady K, et al. Functional Reach Test, Single-Leg Stance Test, and Tinetti Performance-Oriented Mobility Assessment for the prediction of falls in older adults: a systematic review. Phys Ther. 2021;101(10):pzab173.**

**S9. Yang C, Mo Y, Cao X, et al. Reliability and validity of the Tinetti Performance Oriented Mobility Assessment in Chinese community-dwelling older adults. Geriatr Nurs. 2023;53:85-89.**

S10. Hennessey JV, Chromiak JA, DellaVentura S, et al. Growth hormone administration and exercise effects on muscle fiber type and diameter in moderately frail older people. J Am Geriatr Soc 2001;49(7):852-858.

**S11. Sadeghi H, Allard P, Prince F, Labelle H. Symmetry and limb dominance in able-bodied gait: a review. Gait Posture. 2000;12(1):34-45.**

**S12. Perera CK, Gopalai AA, Ahmad SA, Gouwanda D. Muscles affecting minimum toe clearance. Front Public Health. 2021;9:612064.**

S13. Sheng L, Wang XN, Xiao WK, Zhang XY, Li Y, Liu JY, et al. Chinese expert consensus on assessment and management of frailty in the elderly with cardiovascular diseases. Chin J Mult Organ Dis Elderly 2023;22(7):481-91.
